# Mycobacterial mannose-capped lipoarabinomannan: a modulator bridging innate and adaptive immunity

**DOI:** 10.1080/22221751.2019.1649097

**Published:** 2019-08-03

**Authors:** Kai-Liang Zhou, Xin Li, Xiao-Lian Zhang, Qin Pan

**Affiliations:** a State Key Laboratory of Virology and Medical Research Institue, Hubei Province Key Laboratory of Allergy and Immunology and Department of Immunology, Wuhan University School of Medicine, Wuhan, People’s Republic of China; b The eighth hospital of Wuhan, Wuhan, People’s Republic of China

**Keywords:** *M. tb*, ManLAM, innate and adaptive immunity, immunomodulatory, lipoglycan, infection

## Abstract

Mannose-capped lipoarabinomannan (ManLAM) is a high molecular mass amphipathic lipoglycan identified in pathogenic *Mycobacterium tuberculosis* (*M. tb*) and *M. bovis* Bacillus Calmette-Guérin (BCG). ManLAM, serves as both an immunogen and a modulator of the host immune system, and its critical role in mycobacterial survival during infection has been well-characterized. ManLAM can be recognized by various types of receptors on both innate and adaptive immune cells, including macrophages, dendritic cells (DCs), neutrophils, natural killer T (NKT) cells, T cells and B cells. MamLAM has been shown to affect phagocytosis, cytokine production, antigen presentation, T cell activation and polarization, as well as antibody production. Exploring the mechanisms underlying the roles of ManLAM during mycobacterial infection will aid in improving tuberculosis (TB) prevention, diagnosis and treatment interventions. In this review, we highlight the interaction between ManLAM and receptors, intracellular signalling pathways triggered by ManLAM and its roles in both innate and adaptive immune responses.

## Introduction

Tuberculosis (TB), predominately caused by *Mycobacterium tuberculosis* (*M. tb*) is a leading cause of death worldwide [1]. Approximately one third of the world’s population is infected with *M. tb*; however, fewer than 10% of *M. tb*-infected individuals will develop clinical disease. The different outcomes of exposure to *M. tb* predominantly depend on the interaction between the invading bacteria and the host immune system [2]. Both innate and adaptive immunity are essential for protecting the host against *M. tb* infection.

The innate immune response to *M. tb* is initiated upon recognition of its unusual lipid-rich cell wall*.* The mycobacterial cell wall is mainly comprised of mycolic acids (long chain fatty acids), glycolipids and lipoglycans [3]. Mannose-capped lipoarabinomannan (ManLAM) is a high molecular mass amphipathic lipoglycan identified in *M. tb*, *Mycobacterium leprae*, *Mycobacterium bovis* (*M. bovis*), Bacillus Calmette-Guérin (BCG), as well as *Mycobacterium avium* complex [4]. ManLAM has a tripartite structure including a mannosyl-phosphatidyl-*myo*-inositol anchor (MPI), a polysaccharide backbone composed of D-mannan and D-arabinan, and various mannose-capping motifs [5].

The biological functions of ManLAM during *M. tb* infection remain unknown because of the complexity and heterogeneity of the ManLAM structure. As a prominent pathogen-associated molecular pattern (PAMP) and antigen of *M. tb*, ManLAM is recognized by numerous cell receptors, and interacts with several types of innate and adaptive immune cells. In this review, we highlight the interaction between ManLAM and receptors, intracellular signalling pathways triggered by ManLAM and its role in immunomodulation of both innate and adaptive immunity.

## ManLAM structure

**Figure 1.**
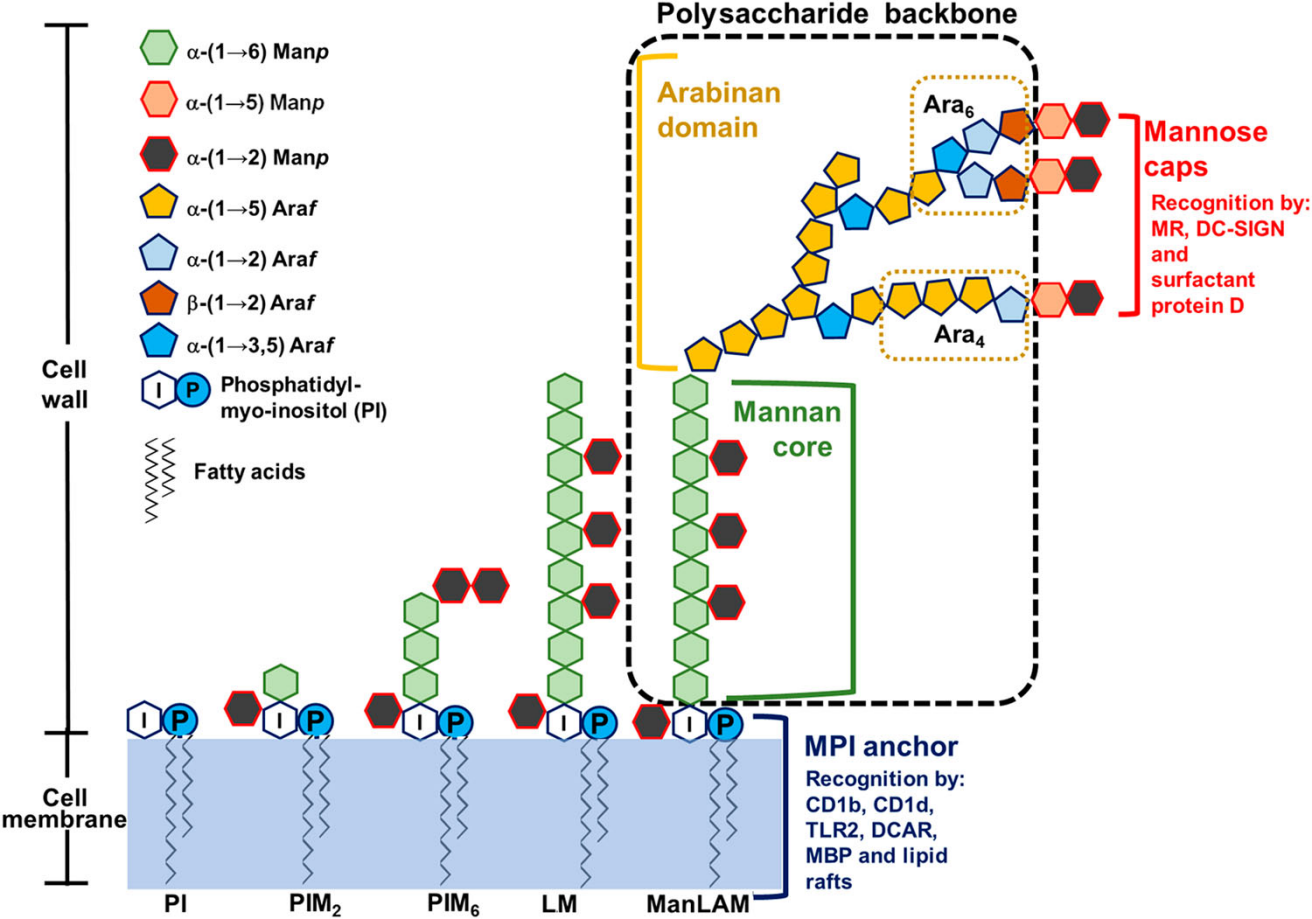
ManLAM structure. ManLAM biosynthesis follows a pathway from phosphatidyl-*myo*-inositol (PI)→PIM→LM→LAM→ManLAM. ManLAM contains three domains: an MPI anchor, a polysaccharide backbone and mannose caps. The MPI anchor comprises a PI unit with Man*p* units. PI acts as an anchor inserted into the cell membrane. The MPI anchor is recognized by CD1b, CD1d, TLR2, DCAR, MBP and lactosylceramide enriched lipid rafts. The polysaccharide backbone includes a mannan core and an arabinan domain. In the mannan backbone of LAM/ManLAM, PIM_2_ is linked to another 17–19 residues of Man*p*. The arabinan core consists of a branched linear α (1→5) linked Ara*f*. Mature LAM/ManLAM is further linked *via* an arabinan domain made up of approximately 70 Ara*f* residues. Two arrangements or motifs can be found at the non-reducing end: a branched hexaarabinofuranoside (Ara_6_) and a linear tetraarabinofuranoside (Ara_4_). The mannose caps consist of one to three Manp residues linked to the terminal *β*-linked Ara*f* unit. The mannose caps are recognized by MR, DC-SIGN and surfactant protein D.

ManLAM consists of three domains (Figure 1): an MPI anchor, a polysaccharide backbone and mannose caps. The MPI anchor is based on an *sn*-glycero-3-phospho-(1-D-*myo*-inositol) unit with α-D-mannopyranosyl (Man*p*) units linked at *O*-2 and *O*-6 of the *myo*-inositol [3]. The polysaccharide backbone includes a mannan core and an arabinan domain. The mannan core consists of α-(1→6) linked mannopyranosyl residues, and at some points α-(1→2) substituted with additional mannopyranosyl motifs. The arabinan domain consists of α-(1→5) linked arabinofuranosyl (Ara*f*) residues with an additional linear α-(3→5) linked Ara*f*. A branched hexaarabinofuranoside (Ara_6_) or a linear tetraarabinofuranoside (Ara_4_) can be found at its non-reducing end. The mannose caps consist of one to three Man*p* residues linked to the terminal *β*-linked Ara*f* unit. The first Man*p* residue is α-(1→5) linked to the terminal *β*-linked Ara*f* unit, and the following Man*p* residues of the caps are α-(1→2) linked to each other. However, capping is a non-stoichiometric process. The degree of mannose capping in ManLAM varies according to the different *M. tb* strains, and the number of mannose residues per cap also varies, even within ManLAM from the same *M. tb* strain [4].

## ManLAM recognition and signalling pathways

ManLAM can be recognized by multiple receptors and soluble molecules because of its structural complexity. The mannose caps of ManLAM are recognized by mannose receptor (MR), the dendritic cell-specific intercellular adhesion molecule-3-grabbing non-integrin (DC-SIGN) and surfactant protein D (Figure 1) [6–9]. The MPI anchors of ManLAM are recognized by CD1 (including CD1b and CD1d), Toll like receptor 2 (TLR2), dendritic cell immunoactivating receptor (DCAR), mannose-binding protein (MBP), and lactosylceramide enriched lipid rafts in the plasma membrane (Figure 1) [10–16]. Other receptors and molecules that bind to ManLAM include pulmonary surfactant protein A (PS-A), Dectin 2 and CD44 [17–21], however, the ManLAM binding domains that interact with these molecules remain unknown.

**Figure 2.**
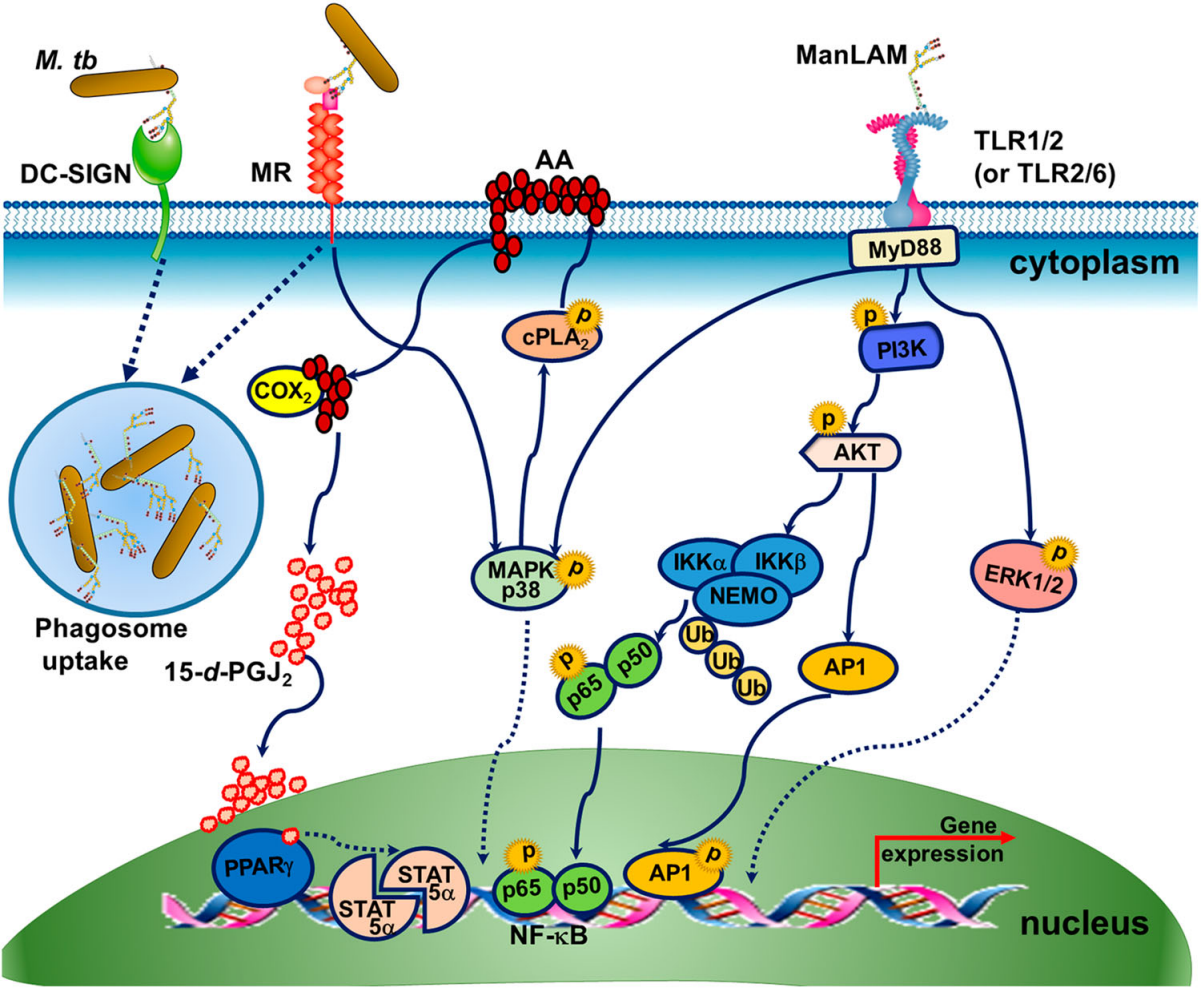
The signalling pathway induced by ManLAM. (1) Recognition of ManLAM by MR and DC-SIGN mediates the phagocytosis of *M.tb*. (2) MR mediates the enhanced expression of the transcription regulatory factor PPAR-γ and the subsequent upregulation of transcription of the signal transducer and activator of transcription (STAT)-5*α*. ManLAM binding to MR leads to activation of MAPK-p38-mediated cytosolic phospholipase A_2_ (cPLA_2_), which drives the release and hydrolysis of arachidonic acids (AA) from the plasma membrane to generate the ligand (15-deoxy-Δ12,14 PGJ_2_, 15-*d*-PGJ_2_) for PPAR*γ*. PPAR*γ* activation leads to the activation of STAT-5*α*. (3) The TLR2 signalling pathway is triggered by ManLAM. ManLAM recognition by TLR1/2 and TLR2/6 drives the MyD88-dependent pathway. MyD88 activation results in activation of NF-κB, AP-1, MAPK p38 and ERK1/2. Phosphorylation of PI3 K and AKT is required for NF-κB and AP-1 activation. Lys48 (K48) -linked ubiquitination of NEMO is also involved in the upstream signal transduction of NF-κB.

Compared with studies examining ManLAM receptors, limited studies have addressed the intracellular signalling pathways triggered by ManLAM. Recognition of ManLAM by MR and DC-SIGN mediates the phagocytosis of *M.tb* by macrophages and dendritic cells (DCs)(Figure 2) [22,23]. Although the cytoplasmic domain of MR does not contain any signalling motifs, MR has been shown, together with other receptors, to participate in intracellular signalling leading to target gene expression (Figure 2) [24]. ManLAM binding with MR mediates enhanced expression of the transcription regulatory factor peroxisome proliferator-activated receptor-gamma (PPAR-γ) and the subsequent transcription upregulation of signal transducer and activator of transcription (STAT)-5*α* [25–27]. Increased PPAR-γ may be associated with ManLAM-dependent inhibition of macrophage apoptosis, reduction of nitric oxide (NO) and oxygen radicals and limiting proinflammatory cytokine production (Figure 2) [25–27].

TLR2 usually dimerizes with TLR1 or TLR6 to recognize lipoglycans and lipoglycoproteins [12,13]. Both TLR1/2 and TLR2/6 have been shown to bind with ManLAM and subsequently trigger the MyD88-dependent signalling pathway, in which phosphorylation of phosphatidylinositol 3 kinase (PI3 K)/AKT/Ap-1 and Lys48 (K48)-linked ubiquitination of NF-kB are increased (Figure 2) [13]. The engagement of TLR2 by ManLAM also triggers the mitogen-activated protein kinase (MAPK) p38 or ERK1/2 pathway (Figure 2) [28]. The results of the TLR2 signalling pathway are complex, leading to the production of both proinflammatory and anti-inflammatory cytokines [28,29].

## ManLAM and innate immunity

During early *M. tb* infection, ManLAM is recognized by pattern recognition receptors (PRRs), mostly expressed on macrophages and DCs. Once ManLAM is recognized by the cells, these innate immune cells release cytokines to cause inflammation, thus promoting their biological function to eliminate invading bacteria. However, at times these cells are manipulated by *M. tb* to hinder the anti-*M. tb* immune response.

### Cytokine production, polarization and phagosome maturation of macrophages following ManLAM stimulation

Alveolar macrophages play the complex roles during *M. tb* infection. On the one hand, macrophages can develop into M1-polarized cells that engulf and eliminate the invading *M. tb* [30], while on the other hand, macrophages provide a critical intracellular niche that is required for *M. tb* to establish infection [31]. Early studies observed increased tumour necrosis factor (TNF) production by both human and murine macrophages following ManLAM stimulation [32], while later studies demonstrated that ManLAM inhibits various interferon γ (IFN-γ)-mediated microbicidal and tumoricidal activities, and triggers macrophages to produce anti-inflammatory cytokine IL-10 [19,26,33,34]. Previously, we reported that ManLAM recognized by CD44 causes the upregulation of IL-1*β*, IL-12 and iNOS (inducible nitric oxide synthase) expression in macrophages, indicating that ManLAM-CD44 signalling enhances M1 macrophage polarization [19,20]. However, the binding of ManLAM to MR might lead to IL-10 production and impair M1 polarization [19].

**Figure 3.**
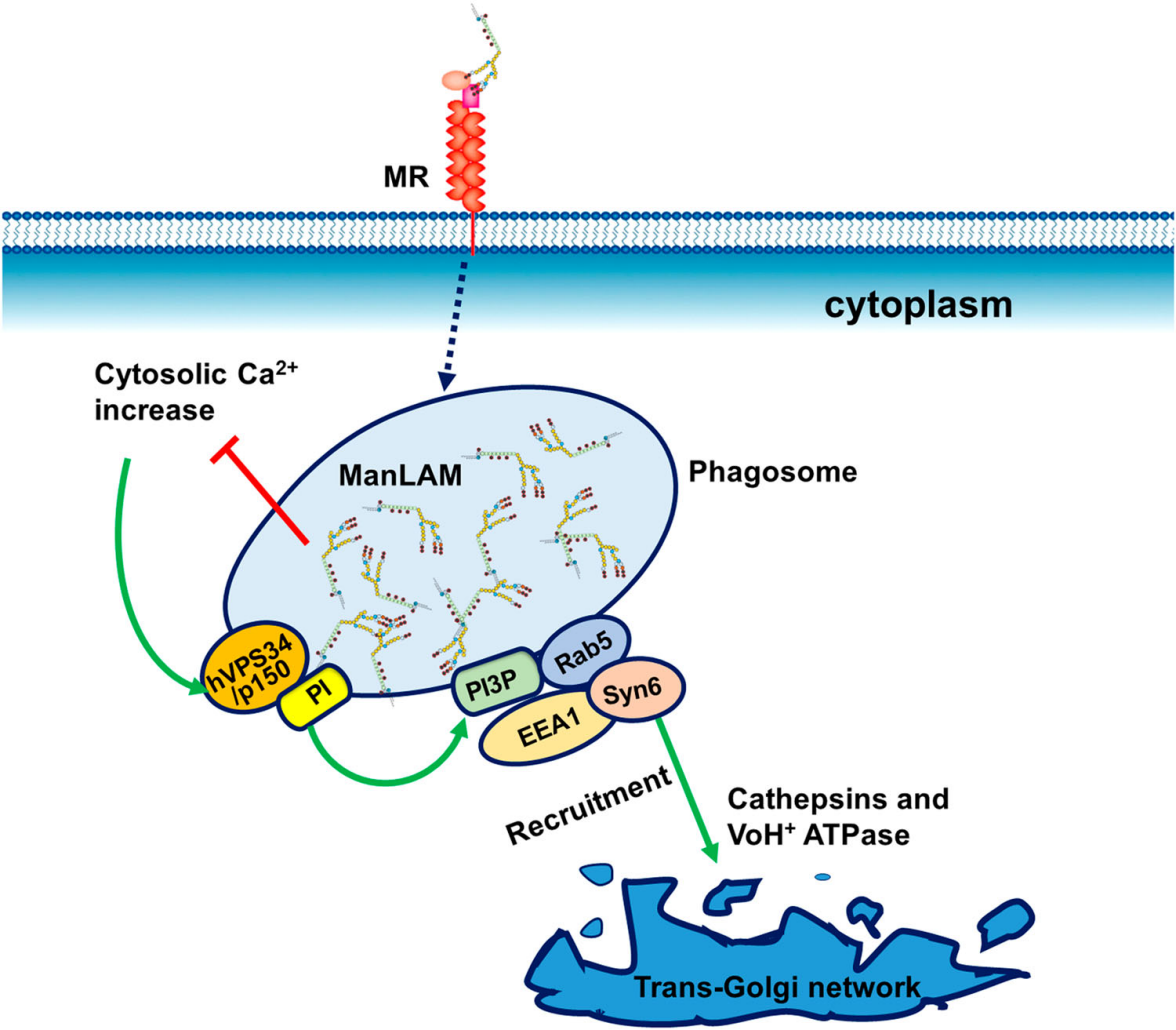
Inhibition of phagosome maturation by ManLAM. The phagosome-lysosome fusion process is classically dependent on cytosolic Ca^2+^ increase. Ca^2+^/calmodulin-dependent PI3-kinase hVPS34 and its modulatory subunit p150 generate phosphatidylinositol 3-phosphate (PI3P) on the phagosomal membrane. PI3P mediates the recruitment of the membrane tethering protein early endosome autoantigen 1(EEA1) to the phagosome. EEA1 is essential for phagosome maturation by directly interacting with syntaxin-6 (Syn6), which is involved in the delivery of cathepsins (lysosomal hydrolases) and VoH1-ATPase from the trans-Golgi network to the phagosome. ManLAM inhibits cytosolic-Ca^2+^ increase and thereby blocks the successive steps, resulting in the prevention of lysosomal fusion and acidification.

Inhibition of phagosome maturation by virulent *M. tb* is an important survival strategy within macrophages, as it allows bacillary replication in the host cells. The binding of *M. tb* ManLAM to MR is involved in inhibition of phagosome maturation [22,35]. After the macrophages phagocytize *M. tb*, ManLAM is released from the bacteria and intercalates into various endomembranes of infected macrophages, which inhibits phagosomal maturation (Figure 3) [36,37]. Moreover, ManLAM inhibits cytosolic-Ca^2+^ increase and subsequently impairs the interaction of PI3 K hVPS34 with calmodulin at the phagosomal membrane, resulting in the arrest of phagosome-lysosome fusion (Figure 3) [38]. Therefore, ManLAM-mediated arrest of phagosome maturation occurs at the stage of late endosomal and lysosomal constituent recruitment [39].

### Activation/maturation and antigen presentation of DCs following ManLAM treatment

The effects that ManLAM exerts on DC activation/maturation may seem contradictory. ManLAM from virulent *M. tb* H37Rv (a potent activator), stimulates the release of TNF, IL-12 and IL-6 and the expression of costimulatory molecules (CD80 and CD86) and antigen-presenting molecules (MHC class II) on human DCs [40]. Subsequently, it has been reported that ManLAM activates DCs *via* Dectin-2 [18]. This recognition causes both pro- and anti-inflammatory cytokine production by DCs, and promotes DC antigen presentation capacity [18]. However, Dulphy et al [41] reported that DCs become partially mature following ManLAM stimulation, as ManLAM-activated DCs display reduced expression of MHC class I and class II molecules, costimulatory molecules (CD83 and CD86) and the chemokine receptor CCR7 compared with lipopolysaccharide (LPS) fully matured DCs.

In contrast, several research groups have suggested that ManLAM inhibits DC maturation. Geijtenbeek et al. [23] demonstrated that ManLAM binding to DC-SIGN interferes with TLR-mediated signals and prevents DC maturation. ManLAM enhances the production of the immunosuppressive cytokine IL-10 in DCs and subsequently downregulates the level of IFN-*γ* from T cells co-cultured with ManLAM-activated DCs [42]. Our group used the ssDNA aptamer ZXL1 to block ManLAM binding to MR, resulting in the reversal of ManLAM-mediated suppression of DC maturation and the enhancement of naïve T cell activation [9]. Although the reason for this discrepancy is unknown, we hypothesize that the different structures of ManLAM from various *Mycobacteria* strains and the preparation methods used contribute to the observed divergent effects on DC maturation [2]. Another prediction is that ManLAM triggers complex signalling pathways in DCs *via* receptors such as Dectin-2, TLRs, DC-SIGN and MR. Specifically blocking the binding of ManLAM to different receptors might cause distinct effects on DC maturation.

### Priming and phagocytosis of neutrophils and activation of NKT cells following ManLAM treatment

Neutrophils can rapidly respond to invading *M. tb*, however, these cells have received relatively little attention in the context of mycobacterial pathogenesis. Fäldt et al [43] reported that ManLAM induces neutrophil priming and an enhanced oxidative response. Nakayama et al [16] described the process of ManLAM-mediated phagocytosis by human neutrophils. While the binding of ManLAM α1,2-monomannose side branches to lactosylceramide (LacCer)-enriched lipid rafts initiates *M. tb* phagocytosis by neutrophils, ManLAM disrupts intracellular signalling and prevents phagolysosome formation [16]. Moreover, ManLAM might modulate neutrophil recruitment *via* DC-SIGN [44]. Blattes et al. [44] showed that oral delivery of either ManLAM or the mimic 3 T mannodendrimer prevents lung inflammation by reducing neutrophil recruitment in mice exposed to aerosolized LPS; this anti-inflammatory effect is dependent on the murine DC-SIGN homolog SIGNR1.

CD1 receptors are a family of lipid-Ag presenting molecules that are structurally related to MHC class I molecules [45]. CD1d presents glycolipids and lipoglycans to CD1d-restricted NKT cells to initiate a rapid innate like response [11]. Upon activation, NKT cells produce IFN-γ, IL-4 and multiple other cytokines and chemokines [46]; and emerging evidence has shown that mouse and human NKT cells may mediate protection against *M. tb* [46,47]. Mycobacterial phosphatidyl-*myo*-inositol mannosides (PIMs) and the more complex lipoarabinomannan (LAM)/ManLAMs potentially induce activation of NKT cells *via* the presentation of these mycobacterial lipoglycans by CD1d [11,48]. Phosphatidylinositol tetramannoside (PIM_4_), the precursor of LAM/ManLAM, is the lipoglycan capable of triggering a distinct population of invariant human (Vα24) and mouse (Vα14) NKT cells [11]. Crystal structure analysis revealed that the hydrophilic CD1d residues interact with the phosphate, inositol and α1-α6-linked mannose of PIM_2_ (the precursor of PIM_4_). Additionally, the α-mannosyl residues at the non-reducing end of LAM/ManLAM have subsequently been shown to be potent activators of mouse (Vα19) NKT cells when they are presented by MR [48]. These results indicate that the MPI anchor in ManLAM might have the ability to activate NKT cells.

## ManLAM and adaptive immunity

Because of the structural complexity of ManLAM, it serves not only as a PAMP molecule recognized by innate immune cells, but is also involved in the adaptive immune response, including CD1b-restricted T cell activation, interference with TCR signalling, induction of regulatory T cells (Tregs), as well as induction of antibody response and IL-10 producing B (B10) cells.

## T cell activation/polarization and ManLAM

T cells have been shown to recognize ManLAM in the context of the non-polymorphic MHC molecule CD1b [10,11,49]. It has been reported that MR on human macrophages delivers lipoglycan antigens to endosomes for presentation to T cells by CD1b molecules [50]. The MPI anchor of ManLAM (especially acylated chains) is crucial for nonspecifically binding to the hydrophobic CD1b pocket [5,10,51]. Thus, the hydrophilic domains of ManLAM are presented to T cells in the context of CD1b molecules [5,10,51]. Because of ManLAM structural heterogeneity, ManLAMs from different *M. tb* strains show distinct capacity to stimulate CD1b-restricted T cells [51]. Further studies have indicated that a smaller and highly acylated ManLAM MPI anchor facilitates the activation and induction of CD1b-restrited T cell proliferation [10]. Upon encountering mycobacterial lipoglycan antigens, CD1b-restricted T cells become cytotoxic and produce IFN-*γ* and TNF-*α* [52,53], suggesting that these cells may contribute to host defence against *M. tb* infection. For example, Busch et al. [54] reported the presence of a subset of IFN-*γ*-producing ManLAM-CD1b-restricted human CD8^+^T cells in bronchoalveolar lavage cells from donors with latent *M. tb* infection. These CD8^+^T cells express the cytotoxic molecules perforin, granulysin and granzyme B and are responsible for limited *M. tb* growth [54].

Although ManLAM activates CD1b-restricted T cells, it can also hinder the immune response of conventional CD4^+^ T cells (expressing *αβ*TCRs) against *M. tb*. As a lipoglycan, ManLAM directly inserts into CD4^+^T cell membranes and interferes with phosphorylation of ZAP-70, Lck and LAT, leading to inhibition of proximal TCR signalling, and the subsequent induction of T cell anergy *via* genes related to anergy in lymphocytes (GRAIL)[50,55]. Proteomics and network analysis revealed that ManLAM induces global changes in the CD4^+^ T cell proteome by affecting Akt-mTOR signalling, resulting in broad functional impairment of CD4^+^ T cell activation [56]. Moreover, ManLAM is indirectly involved in the induction of CD4^+^CD25^+^FoxP3^+^ Tregs. ManLAM stimulates monocytes to produce prostaglandin E2, which subsequently promotes Treg expansion [57]. The expanded Tregs produce transforming growth factor *β* (TGF-*β*) and IL-10 contributing to the reduction of IFN-*γ*-producing T cell frequencies [58].

In addition to Tregs, ManLAM has been shown to inhibit sphingosine-1-phosphate (S1P)-induced and PI3 K/AKT-dependent migration of Th1 cells, probably contributing to delayed recruitment of Th1 cells into the lungs during mycobacterial infection [59]. The S1P concentration gradient facilitates T cell egress from the lymph nodes and their entrance into the circulation. In human studies, ManLAM has also been shown to decrease type 1 (IL-2 and IFN-γ) and increase type 2 (IL-4 and IL-5) cytokine production by Th cells *in vitro* [60]. Taken together, ManLAM shows inhibitory effects on conventional T cells.

## Antibody and cytokine production by ManLAM-treated B cells

Few studies have addressed the direct impact of *M. tb* ManLAM on B cells. Early studies examing ManLAM effects on B cells focused on anti-LAM antibodies. The antibodies binding to the arabinomannan component of LAM were identified in 1977 [61]. The immunodominant sites on LAM as defined by monoclonal antibodies (mAbs), involve the α linked-D-arabinofuranoside residues [62]. The detailed mechanism underlying the binding specificity of mAb CS-35, originally generated against *M. leprae* LAM, is known [63]. The mAb binds to LAM from various mycobacteria and arabinogalactan, and may interact with a linear, terminal oligoarabinofuranosyl tetrasaccharide from LAM [63–65]. Anti-LAM antibodies play an important protective role in host immune response to *M. tb* infection in animal models [66,67]. In a human study, Costello *et al*. [68] reported that the human serum IgG response to LAM is associated with a reduced likelihood of *M. tb* dissemination in children, indicating the protective roles of human anti-LAM antibodies.

Further structural analysis of LAM has revealed that LAM is divided into two subtypes, ManLAM and arabinosylated lipoarabinomannan (AraLAM). Higher levels of anti-ManLAM IgG have been observed in patients with active TB infection than the IgG levels in uninfected controls as well as patients with latent TB infection [69]. Human sera containing anti-ManLAM antibodies enhance macrophage microbicidal activity, including opsonization, phagosome acidification and iNOS and NO production, indicating that antibodies mediate multiple biological and protective functions [70]. Two novel human ManLAM-reactive mAbs, IgG A194-01 and IgM P30B9, were isolated by screening *in vitro* cultures of fractionated memory B cells from diagnosed TB patients [12]. A194-01 binds the poly-Ara*f* backbone of ManLAM, while P30B9 binds with specific capping motifs at the non-reducing termini [71].

In addition to antibody production, ManLAM appears to upregulate CD40 expression and modulate cytokine production. CD40, a costimulatory protein, is expressed on a wide range of cell types including B cells, and the interaction between CD40 and its ligand CD40L is involved in the formation of memory B lymphocytes and promotes immunoglobulin isotype switching. *M. leprae* ManLAM increase CD40 expression on B cells from lepromatous leprosy patients *in vitro*, indicating that *M. leprae* ManLAM could drive the generation of more protective isotype antibodies [72]. Recently, our group reported that ManLAM induces IL-10 production by B cells [13]. We adoptively transferred ManLAM-treated IL-10^-/-^ B cells into *M. tb*-infected Rag2^-/-^ mice, and confirmed that IL-10 produced by ManLAM-induced B cells facilitate mycobacterial survival *in vivo* [13]. Collectively, B cells stimulated with ManLAM have both positive and negative effects on anti-*M. tb* immunity. ManLAM can induce B cells to produce the protective antibodies, while ManLAM causes B cells to produce anti-inflammatory cytokine IL-10 that hinders the immune response to some extent.

## Conclusion

ManLAM, as both an immunogen and a modulator, will aid in improving tuberculosis (TB) prevention, diagnosis and treatment interventions. In the TB diagnostics field, soluble ManLAM secreted from *M. tb* and infected cells is an important immunodiagnostic target. Using antibodies and aptamers, *M. tb* ManLAM can be detected in blood, urine and sputum from TB patients [73–75]. In the field of TB prevention and treatment, selective blockage of the immunoinhibitory pathway mediated by ManLAM might provide an attractive strategy. We have generated the anti-ManLAM aptamer BM2, which blocks ManLAM binding to MR on macrophages, but does not interfere with the ManLAM-CD44 interaction [19]. The results of *in vitro* and *in vivo* studies demonstrate that the aptamer promotes macrophage M1 polarization and might serve as a potential adjuvant candidate for the BCG vaccine [19].

However, a number of challenges remain in the field of ManLAM research. The ManLAM synthesis process is closely related to the synthesis of other molecules on *M. tb*. Conflicting results regarding the contribution of ManLAM to mycobacterial virulence have been reported by two research groups that used mutant *M. tb* strains deficient in the mannosyltransferases involved in the ManLAM biosynthesis [76,77]. As multiple enzymes and genes are involved in ManLAM biosynthesis, partially depleting one or several genes might lead to rearrangement that the *M. tb* cell wall. We can not ascertain the real effects of ManLAM during *M. tb* infection based on studies solely dependent on mutant strains.

Additionally, mannosylated cell wall components (including ManLAM, lipomannan (LM) and PIMs) of *M. tb* have the same or similar structures, and these *M. tb* components may compete for the same receptors and/or synergistically interact with several receptors in building the immune response during *M. tb* infection. LM, composed of a mannan core and a MPI, also shows to exert a dual effect on the release of inflammatory cytokines from macrophages [12,78]. A stimulatory effect of LM is mediated through TLR2 signalling, and an inhibitory effect of LM was independent of functional TLR2 [12,78]. Further research findings highlight LM acylation and different pattern recognition receptors involved in this dual effect [79]. Thus, it is not only necessary to assess the effects of ManLAM, but also to comprehensively evaluate the synergistic effects of ManLAM, LM and PIMs, a series of similar molecules.
